# A Label-Free Quantitative Analysis for the Search of Proteomic Differences between Goat Breeds

**DOI:** 10.3390/ani12233336

**Published:** 2022-11-29

**Authors:** Alessio Di Luca, Andrea Ianni, Francesca Bennato, Michael Henry, Paula Meleady, Giuseppe Martino

**Affiliations:** 1 Department of Bioscience and Agro-Food and Environmental Technology, University of Teramo, 64100 Teramo, Italy; 2 National Institute for Cellular Biotechnology, Dublin City University, Dublin 9, D09 DX63 Dublin, Ireland

**Keywords:** goat, biodiversity, muscle, exudate, proteomics, label-free quantification

## Abstract

**Simple Summary:**

Biodiversity conservation is one of the most relevant current issues. The safeguarding of genetic resources of autochthon farm animals at risk of extinction, such as the Teramana goat, reflects the enhancement of the relative animal productions, in order to highlight the peculiarities that can induce breeders to devote themselves to a specific type of breeding and that can attract the interests of potential consumers. Recent advances in proteomics and bioinformatics tools offer the potential to investigate breed differences at the level of proteins (proteome) and consequently in terms of the biological processes and molecular functions of the specific breed under study. However, there is a lack of information on the potentiality of autochthonous breeds such as the Teramana goat. Therefore, in this study, a global differential proteomic experiment was carried out with the purpose of identifying variations in the expression of muscle proteins between the autochthon Teramana goat and the breed commonly used by the industry, Saanen. The results showed a difference in the expression pattern of 41 proteins that clearly separate the two breeds (underlying genetic differences), highlighting biological features that could influence the productivity, resilience and other traits of these animals.

**Abstract:**

The intensification and standardization of livestock farming are causing a decline in the number of animal breeds in many species, such as the goat. The availability of more studies on the potentiality of goat breeds could raise awareness of their importance, conservation and productive possibilities. Label-free quantitative analysis was applied in this study to investigate the proteomic differences between the autochthon Teramana and Saanen goats that could be useful for defining peculiar features of these breeds. A total of 2093 proteins were characterized in the muscle exudate proteome of the Teramana and Saanen breeds. A total of 41 proteins clearly separated the two breeds. Eukaryotic initiation factor proteins and aldehyde-dehydrogenase 7 family-member A1 were up-regulated in the autochthon breed and associated with its resilience, whereas catalase was down-regulated and associated with lower muscular mass. This study is the most detailed report of goat muscle proteome. Several differentially regulated proteins between the two breeds were identified, providing insights into functional pathways that define this organism and its biology.

## 1. Introduction

Domesticated goats, descendants of the wild goat *Capra hircus*, are the most widespread farm animals and among the earliest species to be domesticated by man [1]. This small ruminant can withstand severe conditions and survive by scavenging for food. For these characteristics, goats have often been seen as deleterious for forests and pastures and considered to be marginal animals for sustenance in developing countries in which they have arisen as major farm animals with a continuing rise in number [2,3]. Recently, goats have been gaining in popularity also in industrialized countries, which is probably due to the availability of new research in the field that highlights the nutritional value and healthiness of goat products (e.g., meat and milk that are easier to digest and have positive effects on heart-related diseases) [3]. Modern animal production systems have evolved to optimize economic efficiency; this logic has negatively influenced the genetic variability of many livestock species, including goats, because of the replacement of autochthon breeds with breeds that are more profitable for farms [4]. Yet livestock biodiversity is crucial to maintaining the ability of animal production systems to adapt to global changes [2,5].

Proteomics is the systematic, large-scale analysis of proteins for their identity, quantity and function in a given tissue or fluid. The application of proteomics in animal science follows the trends from human proteomics studies. However, though most of the proteomics methods from human studies can be translated to animal studies, a lack of complete and annotated genome sequences of many animal species is a big challenge, as an underrepresentation of these species in protein sequence databases could be problematic [6]. In recent years, label-free shotgun proteomics has been gaining in popularity in animal science [7,8,9], as it is a reliable, versatile and a cost-effective proteomics approach [10]. This methodology has the ability to discover potentially new and unexpected connections between changes in protein expression and different group comparisons (different breeds, stressors, treatments, etc.). Moreover, it is effective in biomarker discovery due to the capacity of this technology for measuring multiple biomarkers simultaneously. Indeed, biomarkers are likely to be an array of proteins that, in response to a specific stimuli or condition, have a unique expression pattern rather than being one specific protein [10].

Proteomics has been used to compare the proteome of many breeds in different species. In pigs, the proteome of breeds that are commonly used in crossbreeding plans was investigated [7], and a “new pig breed” and an autochthon breed were also compared by proteomics [11]. Similar studies were also carried out in other species such as cattle [12,13] and ovine [14]. Proteomics studies on goats have mainly focused on milk [15,16,17,18], and only a few studies have investigated other substrates such as the muscles [19,20]. For example, Sarah et al. [19] used a gel-based proteomics approach to examine the effect of different heat treatments on goat skeletal muscle proteins. Using a similar approach, Wang et al. [20] studied the difference in the muscle proteomes of three goat breeds with different drip loss values. Moreover, the few proteomics studies investigating the proteome of autochthonous goat breeds were carried out on milk [21,22]. The lack of information on the potentiality of autochthonous breeds is probably one of the main reasons for their substitution with more productive breeds. Indeed, the availability of scientific studies highlighting the characteristics of these breeds and their production could help breeders and society, as well as raise global awareness of their importance and productive possibilities.

Saanen goats originate from the Saanen Valley in Switzerland. This breed is one of the most widely distributed in the world for the upgrading of local goats to increase milk and meat production. This breed has a high reproductive index, multi parturition, high milk production and high growth and puberty rates [23]. The Teramana goat is an autochthonous breed from the Abruzzo region of Italy that has adapted to the local environment (hills/mountains). Unfortunately, not many studies are available on this breed and, according to the official national data of the Food and Agriculture Organization of the United Nations (FAO), the Teramana goat is listed as a breed with a critical risk status of extinction [24]. The goat breeds under study have different phenotypic characteristics, such as different weights and body sizes, meat and fiber production and hair types and colors. Moreover, they have different capacities to adapt to mutable environmental conditions and husbandry, etc., differences that underly genetic variation. The genetic characterization of breeds is pivotal in order to obtain useful data that could be applied in many applications, such as the management of animal genetic resources, the assessment of inbreeding, breed prioritization for conservation, etc. [25]. Proteomics completes and extends the study of the genome and transcriptome, reflecting the biochemical outcome of genetic information [26]. In the present study, quantitative proteomic analysis was performed to unravel the differences in muscle exudate proteins between the autochthon Teramana goat and the breed commonly used by the industry, Saanen. The final aim of this study was to characterize proteins that can reflect breed genetic differences that could be useful for describing specific features of the breeds and also be applied in the management of this species.

## 2. Materials and Methods

### 2.1. Animal Welfare Disclaimer

All work involving animals was carried out under the research protocol according to the European legislations (European Union procedures on animal experimentation—Directive 2010/63/EU), as already described [27]. Animals were slaughtered in a local commercial abattoir following standard procedures in accordance with the European Council Regulation 1099/2009 [28].

### 2.2. Sample Collection

Five Teramana and five Saanen male goat kids from different farms were transported at about 50 days of life in a common local farm in Teramo province, Italy, where they were raised. All animals were representative of the two goat breeds and were selected to be homogeneous for age. Standard commercial procedures (handling, feeding, housing and transportation) were used for the handling of the animals in this work [27]. Both goat breeds aged six/seven months were fasted overnight (12 h) before being transported to the abattoir, where goats were electrically stunned and slaughtered. *Longissimus thoracis et lumborum* specimens were collected post-mortem between the 12th and 13th ribs from the left side of each carcass, labeled and then stored on ice until they were processed for protein extraction (exudate).

### 2.3. Exudate Collection

Exudate was collected from the *longissimus thoracis et lumborum* muscle following a slightly modified protocol of Di Luca et al. [29] and Bouton et al. [30] the same day of slaughtering. Briefly, three 5 g cubes (~1 cm^3^; giving a total weight sample of ~15 g) taken from the *longissimus thoracis et lumborum* muscle from each sample were centrifuged for 60 min (Mega Star 3.0, VWR International Srl, Milan, Italy) in a disposable polypropylene centrifuge tube (50 mL; Thermo Fisher Scientific, Waltham, MA, USA). Then, samples (exudate) were snap frozen in liquid nitrogen and stored (−80 °C). Using this method, an amount of exudate ranging from about 20 μL to 180 µL (average 60 μL) was collected. The Bradford method (Bio-Rad Protein Assay Kit (Bio-Rad Labs, Hercules, CA, USA)) was used to determinate the protein concentrations of the muscle exudate samples (in triplicate and using a BSA standard) [31]. The concentrations were found to be between 39 and 85 μg/μL.

### 2.4. Filter-Aided Sample Preparation (FASP)

A total of 100 μg of muscle exudate protein samples (five biological replicates (one sample from each animal) for each breed (Teramana and Saanen goats)) were used for the filter-aided sample preparation (FASP) method [32,33]. Briefly, protein samples (100 μg of muscle exudate proteins) were suspended in 100 μL of lysis buffer (7M Urea (Affymetrix/Thermo Fisher Scientific, Waltham, MA, USA), 2M Thiourea (Affymetrix/Thermo Fisher Scientific, Waltham, MA, USA), 30 Mm Tris, 4% CHAPS (Affymetrix/Thermo Fisher Scientific, Waltham, MA, USA), pH 8.5). Dithiothreitol (DTT) and iodoacetamide (IAA; Sigma-Aldrich/Merck, Saint Louis, MO, USA) were used to reduce and alkylate the muscle exudate protein samples, after which trypsin was used for protein digestion according to the method of FASP [32,33]. C18 spin columns (Thermo Fisher Scientific, Waltham, MA, USA) were used to purify the peptides from each sample, and then peptides samples were dried under vacuum and suspended in a solution of 0.1% trifluoroacetic acid and 2% acetonitrile. These samples were prepared for analysis using LC/MS.

### 2.5. Mass Spectrometry for Label-Free LC/MS

Nano LC–MS/MS analysis was carried out using an Ultimate 3000 nanoRSLC system (Thermo Scientific, Waltham, MA, USA) coupled in-line with an Orbitrap Fusion Tribrid™ mass spectrometer (Thermo Scientific, Waltham, MA, USA). A C18 trap column (C18 PepMap100, 300 μm × 5 mm, 5 μm particle size, 100 Å pore size; Thermo Scientific, Waltham, MA, USA) was loaded with one µL of digest and desalted for 3 min (flow rate of 25 μL/min in 0.1% (*v*/*v*) TFA, 2% (*v*/*v*) acetonitrile (ACN)). The trap column was then switched online with the analytical column (Acclaim PepMap 100, 75 µm × 50 cm, 3 µm bead diameter column; (Thermo Scientific, Waltham, MA, USA)). The following binary gradients were used: (i) solvent A (0.1% (*v*/*v*) formic acid in LC-MS grade water); (ii) solvent B (80% (*v*/*v*) ACN, 0.08% (*v*/*v*) formic acid in LC-MS grade water) using 2–32% B for 75 min, 32–90% B in 5 min and holding at 90% for 5 min at a flow rate of 300 nL/min.

MS1 spectra were acquired over *m*/*z* 400–1500 in the Orbitrap (120 K resolution at 200 *m*/*z*), and automatic gain control (AGC) was set to accumulate 4 × 10^5^ ions with a maximum injection time of 100 ms. Data-dependent tandem MS analysis was performed using a top-speed approach (cycle time of 3 s), and the normalized collision energy was optimized at 35% for collision-induced dissociation (CID). MS2 spectra were acquired with a fixed first *m*/*z* of 100 in the ion trap. The intensity threshold for fragmentation was set to 5000 and included charge states 2+ to 7+. A dynamic exclusion of 50 s was applied with a mass tolerance of 10 ppm. AGC was set to 1 × 10^4^ with a maximum injection time set at 35 ms.

### 2.6. Label-Free LC/MS Quantitative Profiling

The workflow in Proteome Discoverer v.2.1 (Thermo Fisher Scientific, Waltham, MA, USA) with the SEQUEST HT algorithm was used to carry out MS data analysis. Database searches were performed using mass spectrometric files (.raw) searched against the UniProtKB-SwissProt *Capra hircus* database (downloaded February 2020 containing 32,490 sequences) with Proteome Discoverer 2.2 using Sequest HT (Thermo Fisher Scientific, Waltham, MA, USA) and Percolator. The following search parameters were used: (i) 10 ppm peptide mass tolerance; (ii) 0.6 Da MS/MS mass tolerance; (iii) up to two missed cleavages were allowed; (iv) cysteine carbamidomethylation set as a fixed modification; (v) methionine oxidation set as a variable modification. Only highly confident peptide identifications with a false discovery rate (FDR) ≤ 0.01 were considered (identified using a SEQUEST HT workflow coupled with percolator validation in Proteome Discoverer 2.2).

Progenesis QI for proteomics v2.0 software (NonLinear Dynamics, a Waters company, Newcastle upon Tyne, UK) was used for label-free LC–MS analysis as already described by Di Luca et al. [34]. The ANOVA score was used to filter the data obtained (peptides with *p* > 0.05 were eliminated). The MS2 data for the remaining peptides was exported, and the resulting MGF file was used to search the UniProtKB-SwissProt database on the SEQUEST server (www.matrixscience.com accessed on 6 February 2020) for protein identifications. The SEQUEST parameters were as described above. The false discovery rate (FDR) was obtained searching the peptides against a decoy database.

Progenesis QI for proteomics software was used to assign matching features to the peptide identifications. The following criteria were used to consider the proteins identified as differentially expressed: (i) ≥2 peptides matched; (ii) ≥1.5 difference in abundance fold; (iii) ANOVA between experimental groups of ≤0.05.

### 2.7. Functional and Protein Network Analyses

The PANTHER (Protein Analysis THrough Evolutionary Relationships) database system, release 14.1 (http://www.pantherdb.org accessed on 15 May 2020) was used for classification analysis of the proteins identified in both groups [35]. The analysis for the categorization into biological processes was carried out using default parameters and the *Bos taurus* genome annotations as the background.

The functional interpretation of the differentially abundant proteins identified was obtained using the ClueGO plug-in (http://www.ici.upmc.fr/cluego) accessed on 15 May 2020) [36] in Cytoscape (http://www.cytoscape.org) [37]. The Gene Ontology (GO)–Biological Process (BP) branch (accessed on 15 May 2020) was used for the gene enrichment analysis. The parameters used were as described in a previous study [7]. Because of insufficient protein annotation for goat species, the analysis made use of *Bos Taurus* specific functional annotations. Default parameters were used for the other parameters. GO:BP terms with a Benjamini–Hochberg corrected *p*-value < 0.05 were considered statistically over-represented. The ClueGO plug-in, which also integrates the KEGG pathway database (accessed on 15 May 2020), was used also to create separate functionally organized pathway term networks.

In silico Protein–Protein Interaction (PPI) analysis of the differentially expressed proteins between goat breeds was carried out using the Search Tool for the Retrieval of Interacting Genes/Proteins (STRING v.11) database (https://string-db.org accessed on 15 May 2020) [11], as already described [7]. The *Bos Taurus* specific interactome was used for the analysis because of insufficient protein annotation for goat species. The analysis considered interactions that had a high confidence (>0.7) STRING combined score.

## 3. Results

### 3.1. Protein Identification in Goat Breeds

A total of 1653 proteins (from 10,038 peptides) were identified and quantified in the Saanen goat bread, whereas 1905 proteins (from 11,744 peptides) were identified and quantified in the Teramana goat (Tables S1 and S2). A total of 2093 proteins were characterized combining these two datasets. A total of 440 (21%) and 188 (9%) proteins were respectively identified in the Teramana and Saanen goat breeds and were unique in each breed. Further, 1465 (70%) proteins were common between the two breeds.

**Figure 1:**
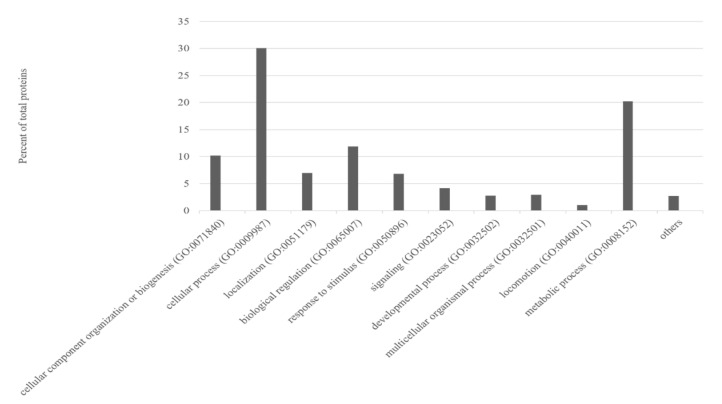
Percentage of proteins (over 2093 identified proteins; Tables S1 and S2) grouped according to different biological processes.

All proteins identified (2093 proteins) underwent gene ontology (GO) analysis through the PANTHER system. Figure 1 shows the categorization of this set of groups into biological processes, indicating that most of these proteins were mainly involved in cellular processes (30.1%), metabolic processes (20.2%), biological regulation (11.9%) and cellular component organization or biogenesis (10.2%).

### 3.2. Label-Free Quantitative Proteomic Analysis of Goat Muscle Exudate

Differential protein abundance was observed between the two goat breeds using Progenesis QI for proteomics with a total of 41 (from 138 peptides) protein pattern changes (*p* ≤ 0.05) between the two goat breeds. Table 1 shows all the 41 proteins identified. Among these proteins, 7 (17.1%) were up-regulated in the Saanen goat, and 34 (82.9%) were up-regulated in the Teramana goat.

### 3.3. Functional Association Analysis

Twenty-one GO:BP terms (25 significantly different proteins) were retrieved (Table 2, Figure S1). Muscle exudate proteins in both breeds were involved primarily in cellular and metabolic process, in biological regulation and response to stimuli. As shown in Table 2 and Figure S1, the most common biological processes were the negative regulation of proteasomal ubiquitin-dependent protein catabolic process (13.04%); the interferon-gamma-mediated signaling pathway (12.50%); the type I interferon signaling pathway (10.53%); cellular response to interleukin-4 (9.09%); the positive regulation of viral genome replication (7.14%).

Table 3 and Figure S2 show KEGG pathway analysis of all the significantly different proteins in goat muscle exudate. We obtained a total of 25 KEGG pathways, and 20 proteins were involved in these pathways. Most of these proteins fell under cell signaling, metabolism- and disease-related pathways, showing mainly a higher concentration in the Teramana goat breed.

### 3.4. Protein–Protein Interaction (PPI) Analysis

**Figure 2:**
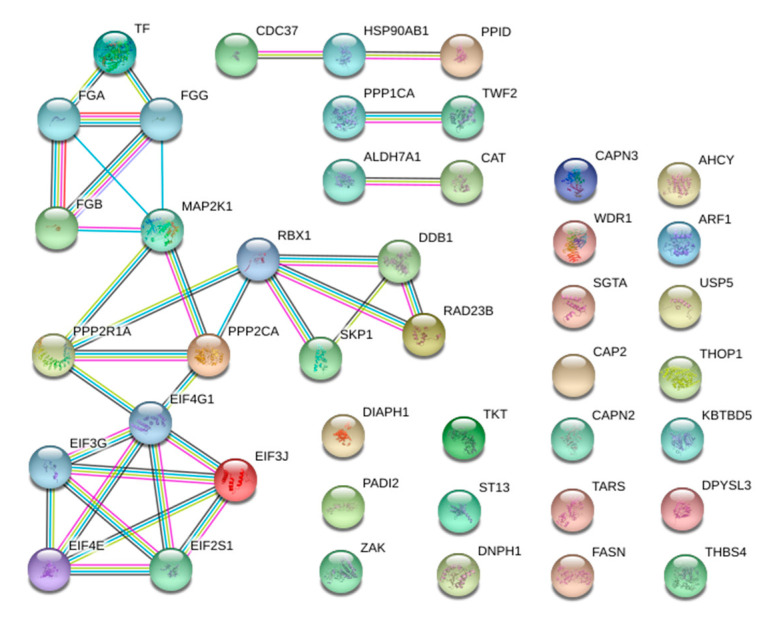
Protein–protein interaction (PPI) network of the proteins characterized between the Teramana and Saanen goat breeds generated with STRING software. Each node represents a protein, and different line colors represent the types of evidence for the association: cyan is from curated databases, magenta is experimentally determined, dark green is gene neighborhood, red is gene fusions, blue is gene co-occurrence, light green is textmining, black is coexpression and purple is protein homology.

Figure 2 show the PPI analysis for the 41 differentially abundant proteins in the two goat breads. The PPI network of muscle exudate contain 43 nodes and 34 edges (vs. 16 detected edges), with a PPI enrichment *p*-value of 7.61 × 10^−5^. The 43 nodes are divided in one big module composed of 16 nodes (37.2%), one small module composed of three nodes (7%), two small components of two proteins (9.3%) and 20 singletons (46.5%). The majority of the proteins in this network interacted with only one or two other proteins (node degree average: 1.58). The big module shows a cluster of five eukaryotic initiation factor (EIF) proteins (EIF4G1, EIF3J, EIF3G, EIF4E and EIF2S1). EIF4G1 showed the highest degree of connection (six edges), whereas the other four EIFs proteins presented four edges, which may assign to them a centrality role in the network. EIFs identified had a higher expression in the Teramana goat breed and were included in the GO and KEGG analyses showing a clear differentiation between the two goat breeds.

## 4. Discussion

In the last decades, despite the strong relationship between livestock production, biodiversity and ecosystem services, a decrease has been observed in the number of livestock breeds in many species, including small ruminants such as goats, with standardization and intensification of animal production systems being the main cause of this [38]. Label-free proteomics profiling was applied in our study with a view to identifying the peptides and proteins indicative of two goat breeds, the autochthon Teramana goat and the goat breed commonly used by the industry, Saanen. Deeper studies of the biochemical process taking place in these breeds improve our knowledge of the genetic and phenotypic differences between them. Moreover, investigating these processes in a matrix that is easy to prepare may lead to possible industrial applications for breed-specific biomarkers.

Only a few proteomics studies have been carried out in goat muscle. These were mainly carried out using gel-based proteomics approaches, and identifying, in most cases, few proteins/protein spots. For example, Sarah et al. [19] used 2DE to make three proteomic maps of goat muscle subject to different heat treatments: chilled, boiled and autoclaved. A total of 410 proteins spots were identified, and following MS analysis, 13 proteins were characterized. Using a similar approach, Wang et al. [20] characterized a lower number of protein spots (158) in the muscle proteomic profiles obtained from three goat breeds with different drip loss values, with 22 proteins identified by MS. Recently, Jia et al. [39] used label-free proteomic analysis to identify 289 shared proteins in goat meat samples that were irradiated.

Our label-free proteomics investigation showed 2093 different proteins, most of them taking part in different biological processes such as cellular processes, metabolic processes, biological regulation and cellular component organization or biogenesis. Among the proteins that were identified (e.g., creatine kinase, troponin, peroxiredoxin, triosephosphate isomerase), some proteins were also highlighted in other investigations on meat from goat or other farm animals [19,20,40]. Moreover, protein maps of the skeletal muscle have been made for several farm animals, such as pigs [40,41], cattle [8,42], lamb [43] and also goat [20]. The protein list identified in the present study, which represents by far the broadest picture of the goat muscle exudate proteome, is an improvement on the previous literature and could serve as an impetus for better understanding the biochemical processes in goat meat and could therefore provide novel information about this species and its production. Not many differences were observed comparing the unique proteins identified in each breed. These proteins were involved in the same biological processes, with a higher number of proteins involved in the immune system process in the Teramana breed. Here, several protease were identified, which play pivotal roles in several physiological processes and in innate immunity and contribute to enhancing adaptive immune responses [44]. The higher presence of these proteins could partially explain the better ability of this breed to cope better with severe conditions (e.g., infection).

Using LC-MS/MS profiling, 41 proteins involved in several biological processes were found to be differentially expressed between the Teramana and Saanen goat breeds. These results suggest that the differences among goat breeds can be related to several aspects of the proteome, and that varieties of molecular functions are involved. Such differences at the protein level between breeds of the same species is due to the re-programming of the genome in response to the environment (phenotypic plasticity), allowing for a better adaptation to the different ecological niches [45].

Among the 41 proteins differentially expressed, three were eukaryotic initiation factors (EIFs) proteins, which are involved in the initiation phase of eukaryotic translation. Initiation, elongation, termination and recycling constitute the process of translation. The initiation of mRNA translation is an essential precursory step that influences the protein expression in mammalian cells via the coordination of numerous initiation factors. This is an efficient way for the cell to defeat many stress conditions such as nutrition deficiency, viral infection, etc. [46,47]. Some eIFs proteins, such as eukaryotic translation initiation factor 2 subunit alpha (EIF2S1), are important in the process of stress response [48]. Indeed, during protein translation, the initiation phase of the synthesis of proteins is the phosphorylation of the subunit α of the EIF2S1. This process allows for the preservation of the resources of the cells while a new gene expression program is adopted to prevent stress damage [47]. The skeletal muscle has great mass and capacity to influence metabolism, which for these features has a major influence on the entire body’s metabolic homeostasis, as it is the main site for the storage of nutrients and of glucose utilization [49]. It is known that exercise induces endoplasmic reticulum stress in skeletal muscles, and in this process, the unfolded protein response maintains endoplasmic reticulum proteostasis through translational and transcriptional regulation [50]. The EIF2S1 is one of the proteins that mediates the pathways of the unfolded protein response generated by endoplasmic reticulum stress [51]. Here, an up-regulation of EIF2S1 was observed in the Teramana goat, suggesting that this breed has a higher strength in response to stressors compared to the less rustic goat Saanen. Two other EIFs proteins (eukaryotic translation initiation factor 3 subunit J, EIF3J and eukaryotic translation initiation factor 4E, EIF4E) were identified in this study, and both proteins showed an up-regulation in the Teramana breed. The EIF3 complex includes different core subunits (a to m) that exert different tasks that are not specifically designated to its single subunits during translation initiation [52]. It has been found that some EIF3 subunits could stimulate protein synthesis, playing an important part in protein translation regulation [53] Moreover, many of these subunits have been implicated in cancers via the mis-regulation of their expression and have been implicated in the promotion of the translation of viral proteins [54,55]. A similar role is played by EIF4E that provides a binding site for other regulatory factors such as EIF3 [56]. As previously explained, the Teramana breed has a higher aptitude to adapt to a harsh environment and changing conditions compared to breeds such as Saanen, which could be partially explained by a higher abundance of EIF proteins. Indeed, a rapid adaptation to physiological conditions is required to cope with stressors such as nutrition deficiency, viral infection, etc. EIF proteins are involved in the reprogrammation of the output of the proteome without requiring changes in RNA synthesis, allowing for a fast change in protein levels that in turn allows for a rapid adaptation to physiological conditions of the animal [57,58,59]. Up-regulation of EIF proteins can be linked to desired resistance characteristics to stressors and as such used in breeding strategies that aim to enhance the resilience of this species.

Compared to other kinds of red meat, goat meat is believed to be healthier. Moreover, it contains essential amino acids such as tryptophan, lysine and threonine [60]. In our study, according to the KEGG pathways, two proteins involved in the tryptophan metabolism were identified, aldehyde dehydrogenase 7 family member A1 (ALDH7A1) and catalase (CAT). Aldehyde dehydrogenases (ALDHs) form a large family of isoenzymes that play an important role in cell survival, protection and differentiation via the metabolism and detoxification of aldehydes [61]. ALDH isoenzymes are present in multiple subcellular compartments, including the nucleus, where they may influence gene expression and cellular proliferation [62]. ALDH7A1 protein has been highly conserved throughout evolution and was identified for the first time in plants as an enzyme that was up-regulated during stressors (e.g., dehydration, increased salinity) [63]. This protein plays a key role in mammals during lysine catabolism, whereby it metabolizes α-aminoadipic semialdehyde to its corresponding carboxylic acid, α-aminoadipic acid [64]. Lysine is an essential amino acid, and its metabolism is involved in maintaining the cellular nitrogen pool, the synthesis of glutamate and the formation of ketone bodies [65]. The up-regulation of ALDH7A1 in the Teramana breed, which is linked with the lysine metabolism, is in line with the physiological strategies that allow this breed to better adapt to harsh environments and to survive during times of poor resources of food that could ultimately lead to starvation-induced mortality. Indeed, ketone bodies are produced during ketogenesis to supply energy to organs such as the brain, heart and skeletal muscle under low caloric restriction or fasting. During times of glucose shortage, ketone bodies play an important part in saving glucose utilization and reducing proteolysis [66]. Catalase or hydroperoxidases (CAT) is a heme-containing peroxisomal enzyme common in all living organisms exposed to oxygen that catalyzes the breakdown of hydrogen peroxide (H_2_O_2_) to water and oxygen. CAT is localized to peroxisomes and cytoplasm and is implicated in ethanol metabolism, inflammation, apoptosis, aging and cancer [67]. High levels of H_2_O_2_ and low levels of the antioxidant enzymes, catalase and glutathione peroxidase have been associated with sarcopenic muscle (loss of skeletal muscle). Despite a higher quantity of reactive oxygen species (ROS) and the evidence showing that proteins that are oxidized are associated with sarcopenic muscle, the mitigation of this problem is still uncertain [68]. Indeed, in muscle ageing, the muscle mass is shaped by exercise, but on the other hand, exercise increases free radical generation because of the increment of the aerobic metabolism of the muscles involved. Therefore, it has been speculated that intense exercise is linked with an increase in ROS, which are able to deplete endogenous antioxidants and also to impair biological molecules and important cellular components [69]. The Saanen breed is usually raised intensively, and the up-regulation of CAT observed in our study (about a two-fold change) could be due to the lower muscle exercise that this breed is subject to. The Teramana breed is well-suited for extensive management that facilitates ROS formation and the use of antioxidants such as CAT, and even if this is quite speculative, these qualities could give rise to lower muscular mass of this breed during ageing compared to other breeds raised in intensive farming.

## 5. Conclusions

This study is one of the few that uses label-free proteomic profiling, applying this technology to investigate the muscle proteome of goat breeds. A total of 41 proteins were identified to be significantly different and were able to differentiate the muscle proteomic profile of the autochthon Teramana goat and the goat breed commonly used by the industry, Saanen. These proteomics data constitute a proof of concept of the molecular differences between these goat breeds, in which the proteome matches the genetic and phenotypic differences between breeds, providing insights into functional pathways that define this organism and its biology. The proteins/biomarkers characterized in this study may contribute a step forward to address future breeding, conservation and management policies for this species that is still considered secondary in developed countries but is of particular interest in developing countries.

## Figures and Tables

**Table 1:** Forty-one proteins identified as differentially expressed between the Saanen and Teramana goat breeds following statistical analysis (Progenesis QI for proteomics).

UniProt * (a)	Gene Name	Description	Peptides ^§^	Score ^¥^	Anova (p)	Fold Change	Highest Condition ^‡^
A0A452G990	AHCY	Adenosylhomocysteinase	11	40.71	0.0215	2.96	Saanen
A0A452FKN8	TKT	Transketolase	4	15.06	0.0263	2.02	Saanen
A0A452F4U3	FASN	Fatty acid synthase	3	12.36	0.0289	268.23	Saanen
A0A452ENA3	FGB	Fibrinogen beta chain	2	7.52	0.0330	2.30	Saanen
A0A452FJF9	TF	Transferrin	2	7.25	0.0004	139.18	Saanen
A0A452EBL1	LOC102177638	Aldo_ket_red domain-containing protein	2	8.83	0.0137	1.50	Saanen
A0A452FHG1	CAT	Catalase	2	6.29	0.0263	2.12	Saanen
(b)							
A0A452F327	HSP90AB1	Heat shock protein 90 alpha family class B member 1	10	31.66	0.0022	1.55	Teramana
A0A452G8Z0	CAP2	Adenylyl cyclase-associated protein	10	35.45	0.0292	1.75	Teramana
A0A452G069	WDR1	WD repeat domain 1	7	30.53	0.0156	1.67	Teramana
A0A452FP98	RAD23B	RAD23 homolog B, nucleotide excision repair protein	5	16.07	0.0067	1.53	Teramana
A0A452F4M6	DPYSL3	Dihydropyrimidinase like 3	5	16.07	0.0401	1.59	Teramana
A0A452DWP9	ALDH7A1	Aldehyde dehydrogenase 7 family member A1	4	17.09	0.0089	1.66	Teramana
A0A452FIW7	MAP2K1	Mitogen-activated protein kinase kinase 1	4	11.94	0.0056	1.65	Teramana
A0A452EHA4	TARS	Threonyl-tRNA synthetase	4	12.85	0.0050	1.87	Teramana
A0A452EDQ1	PPP1CA	Serine/threonine-protein phosphatase	4	16.63	0.0079	1.97	Teramana
A0A452FUS0	PPP2CA	Serine/threonine-protein phosphatase	4	14.72	0.0250	1.93	Teramana
A0A452FLI7	EIF3J	Eukaryotic translation initiation factor 3 subunit J	3	9.74	0.0154	1.73	Teramana
A0A452G270	DIAPH1	Diaphanous related formin 1	3	9.94	0.0042	2.49	Teramana
A0A452FYF5	THBS4	Thrombospondin 4	3	8.93	0.0159	2.69	Teramana
A0A452FTI8	PPP2R1A	Protein phosphatase 2 scaffold subunit Aalpha	3	10.30	0.0243	1.51	Teramana
A0A452EMT6	DNPH1	2′-deoxynucleoside 5′-phosphate N-hydrolase 1	3	11.06	0.0268	2.14	Teramana
A0A452ENH1	DDB1	Damage specific DNA binding protein 1	3	7.78	0.0109	1.75	Teramana
A0A452F1P8	ST13	TPR_REGION domain-containing protein	3	18.62	0.0041	1.51	Teramana
A0A452E633	ARF1	ADP ribosylation factor 1	2	9.54	0.0183	1.57	Teramana
A0A452EJ76	SKP1	S-phase kinase-associated protein 1	2	6.79	0.0212	1.52	Teramana
A0A452F1S1	PADI2	Peptidyl arginine deiminase 2	2	4.84	0.0425	2.45	Teramana
A0A452FX49	HBB	Hemoglobin subunit beta-C	2	7.73	0.0457	41.19	Teramana
A0A452DUC4	LOC102176008	Nucleosome assembly protein 1-like 1	2	6.65	0.0198	1.89	Teramana
A0A452EL14	KLHL40	Kelch-like family member 40	2	6.89	0.0347	2.03	Teramana
A0A452ETL2	MAP3K20	Mitogen-activated protein kinase kinase kinase 20	2	7.07	0.0147	1.74	Teramana
A0A452F4J5	CAPN2	Calpain 2	2	7.04	0.0372	2.07	Teramana
A0A452DMC5	CDC37	Cell division cycle 37	2	11.79	0.0115	1.96	Teramana
A0A452E0G5	EIF4E	Eukaryotic translation initiation factor 4E	2	5.93	0.0122	1.97	Teramana
A0A452E5T3	PPID	Peptidyl-prolyl cis-trans isomerase D	2	6.26	0.0024	1.53	Teramana
A0A452E6F8	EIF2S1	Eukaryotic translation initiation factor 2 subunit alpha	2	7.94	0.0062	1.57	Teramana
A0A452EC09	USP5	Ubiquitin carboxyl-terminal hydrolase	2	7.42	0.0273	3.66	Teramana
A0A452G497	SGTA	Small glutamine rich tetratricopeptide repeat containing alpha	2	4.85	0.0152	2.11	Teramana
A0A452EIZ0	THOP1	Thimet oligopeptidase 1	2	6.32	0.0168	2.16	Teramana
A0A452FTG3	CAPN3	Calpain 3	2	6.53	0.0280	2.08	Teramana
A0A452EMB1	TWF2	Twinfilin actin binding protein 2	2	8.27	0.0138	1.91	Teramana

Seven proteins were up-regulated in the Saanen breed, Table 1 (a), and 34 proteins were up-regulated in Teramana goat breed, Table 1 (b). * Accession number in the UniProt database; ^§^ peptides used for quantitation; ^¥^ SEQUEST score; ^‡^ indicates whether the proteins were up-regulated in the Saanen or in the Teramana goat breeds.

**Table 2:** Gene ontology biological process analysis (GO:BP) of differential proteins in the goat breed comparison.

GOID	Description	Functional Group *	*p*-Value ^§^	% of Associated Proteins ^¥^	N. of Proteins	Up or Down Regulated Proteins ^●^
GO:0008064	regulation of actin polymerization or depolymerization	G0	0.008	2.27	3	ARF1 ↓, TWF2 ↓, WDR1 ↓
GO:0045070	positive regulation of viral genome replication	G1	0.005	7.14	2	DDB1 ↓, PPID ↓
GO:0010038	response to metal ion	G2	0.010	2.00	3	CAPN3 ↓, FGB ↑, TF ↑
GO:0014902	myotube differentiation	G3	0.021	2.38	2	CAPN2 ↓, KLHL40 ↓
GO:0017148	negative regulation of translation	G4	0.006	2.52	3	EIF2S1 ↓, EIF4E ↓, MAP2K1 ↓
GO:0032436	positive regulation of proteasomal ubiquitin-dependent protein catabolic process	G5	0.015	3.03	2	SGTA ↓, USP5 ↓
GO:0045214	sarcomere organization	G6	0.007	5.88	2	CAPN3 ↓, WDR1 ↓
GO:0051146	striated muscle cell differentiation	G7	0.004	2.07	4	CAPN2 ↓, CAPN3 ↓, KLHL40 ↓, WDR1 ↓
GO:0051781	positive regulation of cell division	G8	0.010	3.85	2	CAT ↑, THBS4 ↓
GO:0071214	cellular response to abiotic stimulus	G9	0.005	2.11	4	CAPN3 ↓, DDB1 ↓, EIF2S1 ↓, PPID ↓
GO:0071353	cellular response to interleukin-4	G10	0.006	9.09	2	FASN ↑, HSP90AB1 ↓
GO:0009416	response to light stimulus	G11	0.002	2.33	5	CAT ↑, DDB1 ↓, EIF2S1 ↓, PPID ↓, PPP1CA ↓
GO:1990266	neutrophil migration	G12	0.020	2.50	2	THBS4 ↓, WDR1 ↓
GO:0001959	regulation of cytokine-mediated signaling pathway	G13	0.006	4.17	3	CDC37 ↓, HSP90AB1 ↓, PADI2 ↓
GO:0060333	interferon-gamma-mediated signaling pathway	G13	0.006	12.50	2	CDC37 ↓, HSP90AB1 ↓
GO:0060337	type I interferon signaling pathway	G13	0.006	10.53	2	CDC37 ↓, HSP90AB1 ↓
GO:0042176	regulation of protein catabolic process	G14	0.001	2.11	6	DDB1 ↓, HSP90AB1 ↓, KLHL40 ↓, RAD23B ↓, SGTA ↓, USP5 ↓
GO:1903320	regulation of protein modification by small protein conjugation or removal	G14	0.001	2.14	4	CAPN3 ↓, HSP90AB1 ↓, KLHL40 ↓, SGTA ↓
GO:0043161	proteasome-mediated ubiquitin-dependent protein catabolic process	G14	0.001	2.40	7	DDB1 ↓, HSP90AB1 ↓, KLHL40 ↓, RAD23B ↓, SGTA ↓, SKP1 ↓, USP5 ↓
GO:0032434	regulation of proteasomal ubiquitin-dependent protein catabolic process	G14	0.001	5.21	5	HSP90AB1 ↓, KLHL40 ↓, RAD23B ↓, SGTA ↓, USP5 ↓
GO:0032435	negative regulation of proteasomal ubiquitin-dependent protein catabolic process	G14	0.001	13.04	3	HSP90AB1 ↓, KLHL40 ↓, SGTA ↓

* Groups of closely related terms; ^§^ Benjamini–Hochberg corrected *p*-values; ^¥^ percentage of input proteins found to be associated with respect to the number of proteins directly annotated with the functional term; ^●^ ↑: indicates protein up-regulated in Saanen goat than in Teramana goat; ↓: indicates protein up-regulated in Teramana goat than in Saanen goat.

**Table 3:** KEGG pathway analysis of differential proteins in the goat breed comparison.

GOID	Description	Functional Group *	*p*-Value ^§^	% of Associated Proteins ^¥^	N. of Proteins	Up or Down Regulated Proteins ^●^
KEGG:00380	Tryptophan metabolism	G0	0.020	4.35	2	ALDH7A1 ↓, CAT ↑
KEGG:04211	Longevity regulating pathway	G1	0.044	2.17	2	CAT ↑, EIF4E ↓
KEGG:05143	African trypanosomiasis	G2	0.022	4.44	2	HBB ↓, THOP1 ↓
KEGG:05144	Malaria	G3	0.023	3.33	2	HBB ↓, THBS4 ↓
KEGG:05160	Hepatitis C	G4	0.020	2.10	3	EIF2S1 ↓, PPP2CA ↓, PPP2R1A ↓
KEGG:04066	HIF-1 signaling pathway	G5	0.012	3.03	3	EIF4E ↓, MAP2K1 ↓, TF ↑
KEGG:04114	Oocyte meiosis	G6	0.001	4.17	5	MAP2K1 ↓, PPP1CA ↓, PPP2CA ↓, PPP2R1A ↓, SKP1 ↓
KEGG:04140	Autophagy	G7	0.019	2.22	3	EIF2S1 ↓, MAP2K1 ↓, PPP2CA ↓
KEGG:04141	Protein processing in endoplasmic reticulum	G8	0.002	3.01	5	CAPN2 ↓, EIF2S1 ↓, HSP90AB1 ↓, RAD23B ↓, SKP1 ↓
KEGG:04152	AMPK signaling pathway	G9	0.022	2.36	3	FASN ↑, PPP2CA ↓, PPP2R1A ↓
KEGG:04210	Apoptosis	G10	0.019	2.05	3	CAPN2 ↓, EIF2S1 ↓, MAP2K1 ↓
KEGG:04218	Cellular senescence	G11	0.010	2.33	4	CAPN2 ↓, MAP2K1 ↓, PPID ↓, PPP1CA ↓
KEGG:04350	TGF-beta signaling pathway	G12	0.009	3.61	3	PPP2CA ↓, PPP2R1A ↓, SKP1 ↓
KEGG:04510	Focal adhesion	G13	0.004	2.46	5	CAPN2 ↓, DIAPH1 ↓, MAP2K1 ↓, PPP1CA ↓, THBS4 ↓
KEGG:04720	Long-term potentiation	G14	0.027	2.90	2	MAP2K1 ↓, PPP1CA ↓
KEGG:04910	Insulin signaling pathway	G15	0.006	2.80	4	EIF4E ↓, FASN ↑, MAP2K1 ↓, PPP1CA ↓
KEGG:03420	Nucleotide excision repair	G16	0.022	4.44	2	DDB1 ↓, RAD23B ↓
KEGG:04071	Sphingolipid signaling pathway	G17	0.020	2.48	3	MAP2K1 ↓, PPP2CA ↓, PPP2R1A ↓
KEGG:04730	Long-term depression	G17	0.020	5.00	3	MAP2K1 ↓, PPP2CA ↓, PPP2R1A ↓
KEGG:04914	Progesterone-mediated oocyte maturation	G18	0.170	2.15	2	HSP90AB1 ↓, MAP2K1 ↓
KEGG:04915	Estrogen signaling pathway	G18	0.170	2.00	2	HSP90AB1 ↓, MAP2K1 ↓
KEGG:05215	Prostate cancer	G18	0.170	2.08	2	HSP90AB1 ↓, MAP2K1 ↓
KEGG:03015	mRNA surveillance pathway	G19	0.082	3.06	3	PPP1CA ↓, PPP2CA ↓, PPP2R1A ↓
KEGG:04261	Adrenergic signaling in cardiomyocytes	G19	0.082	2.07	3	PPP1CA ↓, PPP2CA ↓, PPP2R1A ↓
KEGG:04728	Dopaminergic synapse	G19	0.082	2.24	3	PPP1CA ↓, PPP2CA ↓, PPP2R1A ↓

* Groups of closely related terms; ^§^ Benjamini–Hochberg corrected *p*-values; ^¥^ percentage of input proteins found to be associated with respect to the number of proteins directly annotated with the functional term; ^●^ ↑: indicates protein up-regulated in Saanen goat than in Teramana goat; ↓: indicates protein up-regulated in Teramana goat than in Saanen goat.

## Data Availability

Not applicable.

## Supplementary Materials

The following supporting information can be downloaded at: https://www.mdpi.com/article/10.3390/ani12233336/s1, Table S1. List of the 1653 proteins characterized by mass spectrometry in the goat muscle exudate in the Saanen goat bread determined in Proteome Discoverer using SEQUEST HT algorithm. MS files were searched against the UniProtKB-SwissProt *Capra hircus* database (06 February 2020 containing 32,490 sequences). ^(a)^ Total number of the peptide sequences that were identified for the protein, including those redundantly identified. ^(b)^ Number of peptide sequences that were unique to a protein group. ^(c)^ Theoretical molecular weight. Table S2. List of the 1905 proteins characterized by mass spectrometry in the goat muscle exudate in the Teramana goat bread determined in Proteome Discoverer using SEQUEST HT algorithm. MS files were searched against the UniProtKB-SwissProt *Capra hircus* database (06 February 2020 containing 32,490 sequences). ^(a)^ Total number of the peptide sequences that were identified for the protein, including those redundantly identified. ^(b)^ Number of peptide sequences that were unique to a protein group. ^(c)^ Theoretical molecular weight. Figure S1. Gene ontology biological process analysis (GO:BP) of the differentially expressed proteins of the Teramana and Saanen goat breeds. Bars show the percentage of the proteins that were associated with respect to the number of proteins directly annotated with the functional term. The term’s significance and the number of proteins related to the term are reported next to each bar. Table 2 show the statistics of the analysis. Figure S2. KEGG enrichment of differentially expressed proteins of the Teramana and Saanen goat breeds. Bars show the percentage of the proteins that were associated with respect to the number of proteins directly annotated with the functional term. The term’s significance and the number of proteins related to the term are reported next to each bar. Table 3 show the statistics of the analysis.
