# Supplementary figures and images for: A Label-Free Quantitative Analysis for the Search of Proteomic Differences between Goat Breeds
Source: Animals (Basel). 2022 Nov 29;12(23):3336. doi: 10.3390/ani12233336 (PMC9740416; doi:10.3390/ani12233336)

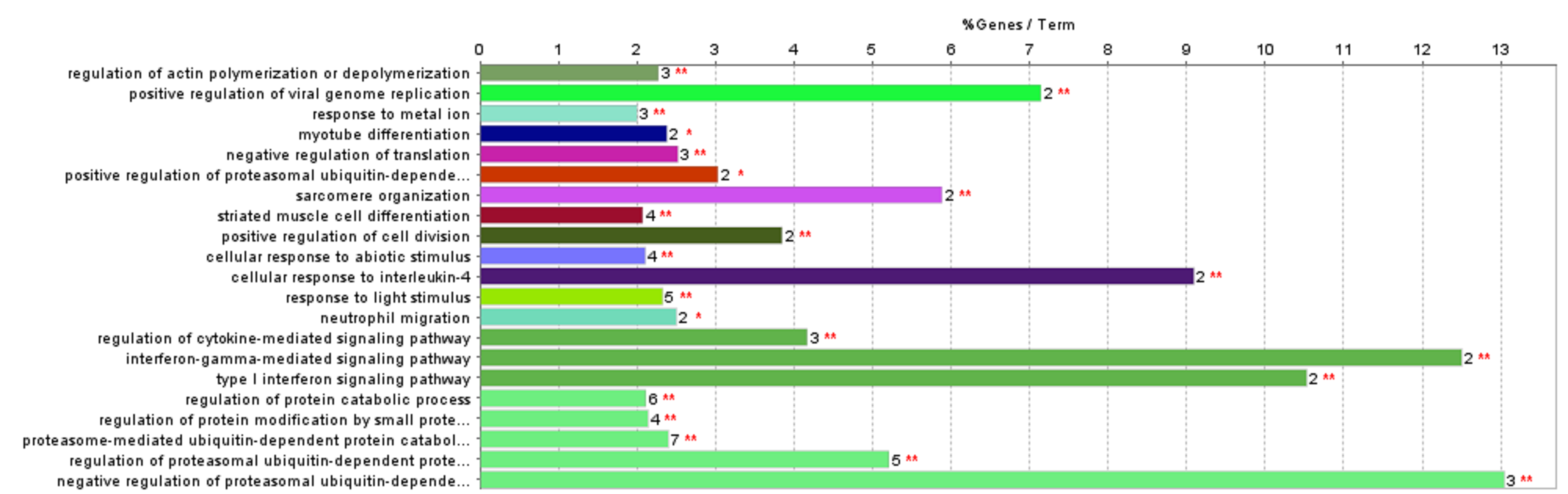

Supplement: Supplementary file 1 [file animals-12-03336-s001.zip › Figure S1.png]

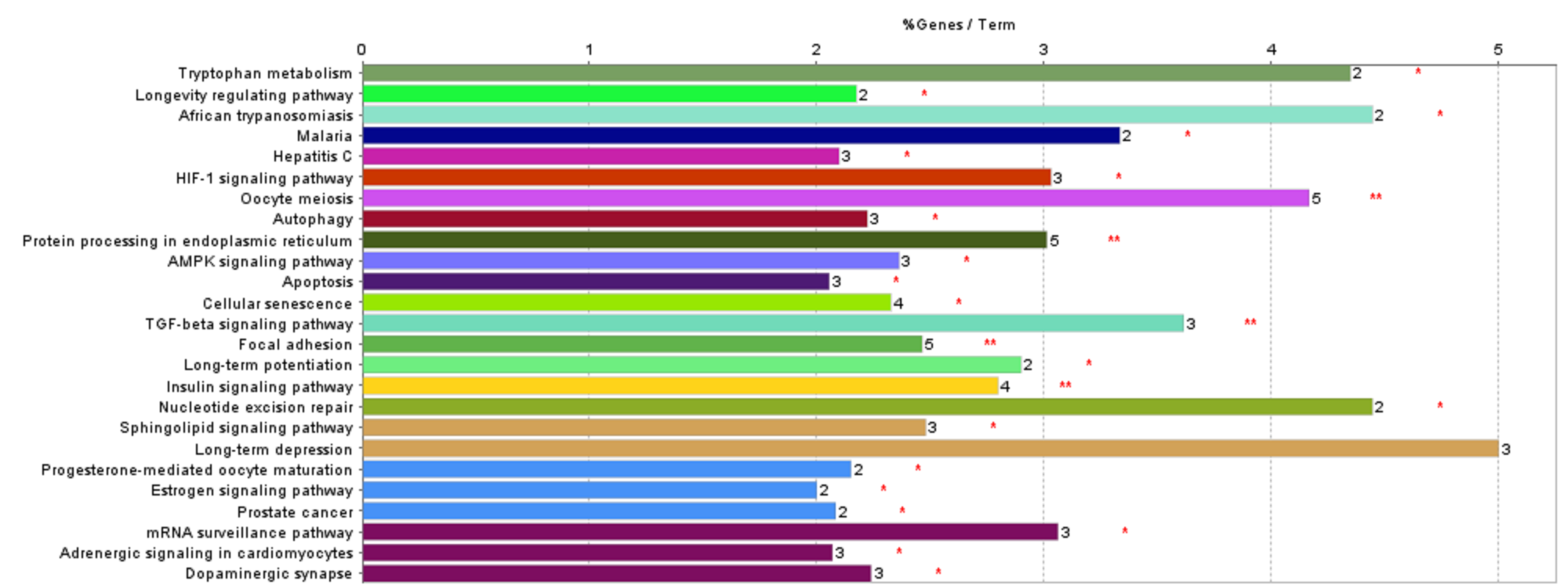

Supplement: Supplementary file 1 [file animals-12-03336-s001.zip › Figure S2.bmp]
